# Serial TIL infusions and PD-1 blockade drive long-term clonal persistence in prostate cancer

**DOI:** 10.3389/fonc.2025.1693912

**Published:** 2025-11-05

**Authors:** Lucas C. M. Arruda, Julia Karbach, Dragan Kiselicki, Kathrin Brand, Claudia Wahle, Evgueni Sinelnikov, Dirk Gustavus, Hans Hoffmeister, Akin Atmaca, Elke Jäger

**Affiliations:** ^1^ Department of Medicine Huddinge, Karolinska Institutet, Stockholm, Sweden; ^2^ CuraCell, Solna, Sweden; ^3^ Department of Oncology and Hematology, Krankenhaus Nordwest, Frankfurt am Main, Germany; ^4^ Zellwerk GmbH, Eichstaedt, Germany

**Keywords:** tumor-infiltrating lymphocytes (TIL), immune checkpoint blockade, pembrolizumab, TCR NGS, anti-PD-1

## Abstract

Adoptive cell therapy using tumor-infiltrating lymphocytes (TIL) can achieve durable responses in patients with metastatic cancers, but the long-term clonal dynamics after multiple administration and synergy with checkpoint blockade remain understudied. We present a longitudinal case study of a patient with treatment-refractory metastatic prostate cancer that achieved complete and durable tumor remission over 5-years after multiple TIL infusions and anti-PD-1 therapy. We performed longitudinal high-throughput T-cell receptor (TCR) sequencing on blood and tumor samples collected over five years to track the persistence and dynamics of TIL-derived and endogenous clonotypes. TIL-derived clonotypes exhibited sustained persistence in blood, with notable clonal expansions correlating with reduced repertoire diversity, increased clonality, and observed clinical response. Multiple TIL administration increased the patient exposure to the therapy, improving its pharmacokinetics profile over time. The third TIL infusion was followed by pembrolizumab administrations, which coincided with the re-expansion of TIL-derived clonotypes and emergence of novel clones. Serial tracking revealed clonotype stability for up to five years post-treatment. Our findings provide insights into the long-term persistence and reactivation of TIL-derived immunity and illustrate the potent synergy between adoptive transfer and PD-1 blockade by enhancing both infused and endogenous tumor-reactive T cell responses, and supporting the integration of longitudinal immunogenomic monitoring in personalized immunotherapy.

## Introduction

Adoptive transfer of tumor-infiltrating lymphocytes (TIL) has become a cornerstone of immunotherapy in advanced melanoma and other solid tumors. A recent review and meta-analysis reported an objective response rate (ORR) of 34% in patients previously treated with immune checkpoint blockade (ICB) and 44% in treatment-naïve melanoma patients (1). In advanced non–small-cell lung cancer (NSCLC), TIL therapy achieved an ORR of 21.4% (2). Responses have also been observed in cervical cancer (44%) (3), head and neck squamous-cell carcinoma (38.9%) (4), and breast cancer (50%) (5), whereas “cold” tumors such as gastrointestinal and ovarian cancers have shown poorer outcomes, ranging from 0% (6, 7) to 16.7% (8). These variable outcomes underscore both the current limitations of TIL therapy in immunologically “cold” tumors and the opportunity to develop strategies that extend its efficacy across a broader range of cancers.

The efficacy of TIL therapy can be further enhanced through tumor-specific T-cell selection protocols and cell engineering techniques (9) or by combining with ICB, with multiple trials currently evaluating different combination strategies (10). In NSCLC, the ORR increased to 64.3% with the addition of anti–PD-1 therapy (11). Similarly, in cervical cancer, TIL monotherapy responses improved to 57.1% when combined with pembrolizumab (4). Synergistic benefits have also been observed in immunologically “cold” tumors, such as chemotherapy-resistant metastatic osteosarcoma, where an ORR of 36.7% was achieved with the addition of nivolumab (12), supporting the potential to broaden the application of TIL therapy to additional hard-to-treat indications.

Mechanistic studies have sought to correlate patient baseline features, tumor characteristics, TIL product profiles, and post-infusion dynamics with clinical responses, aiming to identify biomarkers that optimize efficacy and help overcome resistance (10, 13). Durable outcomes have consistently been linked to the persistence and function of tumor-reactive T-cell clones (13, 14), emphasizing the importance of monitoring immune repertoire dynamics (15). Advances in high-throughput T-cell receptor (TCR) sequencing now allow precise tracking of infused and endogenous T-cell populations, providing insights into their persistence, expansion, and clonal evolution over time (16). Building on this, recent studies suggest that sequential TIL administrations, with or without ICB, can augment antitumor activity by broadening and sustaining T-cell responses (17–22). Similar to repeated CAR-T infusions, which have prolonged remission by preventing antigen escape and maintaining T-cell persistence (23–25), multiple TIL infusions may improve pharmacokinetics and pharmacodynamics by sustaining T-cell activity, promoting deeper tumor infiltration, and enhancing clinical outcomes (21). Beyond strengthening persistence, these strategies may also foster the emergence of new clonotypes, potentially mitigating relapse driven by antigen loss or T-cell exhaustion (17). Nonetheless, the long-term clonal evolution induced by sequential TIL infusions and staggered checkpoint blockade remains poorly defined, particularly in tumor types beyond melanoma.

Here, we present a five-year longitudinal case study of a patient with metastatic castration-resistant prostate cancer (mCRPC) who underwent three sequential TIL infusions, followed by pembrolizumab administration (18). The present follow-up leverages serial TCRβ sequencing of blood and tumor samples to define the long-term fate of infused clonotypes, their redistribution across compartments, and the immunologic consequences of post-TIL PD-1 blockade. This analysis offers a unique, high-resolution view of clonal persistence, diversification, and reactivation in the context of combined adoptive cell transfer and checkpoint inhibition.

## Methods

### Patient and treatment overview

This case study involved a patient with metastatic castration-resistant prostate cancer (mCRPC) treated under compassionate use regulations in Germany (18). The patient received three autologous TIL infusions over a period of six months, followed by anti-PD-1 therapy (pembrolizumab). Each infusion was preceded by lymphodepletion with a single intravenous dose of cyclophosphamide (30 to 60 mg/kg) and followed by low-dose interleukin-2 (600,000 IU/kg; 4 to 5 doses based on tolerance). Clinical responses were assessed using radiographic imaging and RECIST criteria as previously reported (18).

### TIL manufacturing

Tumor-infiltrating lymphocytes (TILs) were expanded from surgical tumor biopsies as previously described (18). In brief, tumor material was processed at Zellwerk GmbH (Berlin, Germany) using a fully closed, GMP-compliant perfusion bioreactor system. Tumor fragments (~8 mm³) were cultured in a 30MM perfusion bioreactor for initial outgrowth (phase 1) and subsequently transferred to a 500MM bioreactor for large-scale expansion (phase 2). Cultures were maintained in GMP-grade CellGenix DC medium supplemented with 10% human serum and 1% antibiotics, under automated control of pH, pO_2_, and temperature. For TIL-1 and TIL-2, cytokine supplementation included IL-2, IL-15, and IL-21, whereas TIL-3 was expanded with IL-2 only during the second phase. Activation was initiated once with anti-CD3 (OKT3) and irradiated allogeneic feeder cells. Final products were washed, formulated in 5% albumin, and released for infusion under ATMP-compliant conditions. TIL-1 and TIL-2 were generated from the same biopsy (June 2018), while TIL-3 was derived from an independent biopsy (January 2019).

### Sample collection

Peripheral blood mononuclear cells (PBMCs) were collected at baseline, post-infusion timepoints (Days +1 to +30), and periodically over five years. Tumor biopsies were obtained prior to the first infusion and approximately two months following the second infusion. Infused TIL products were sampled prior to administration. All specimens were processed and cryopreserved using standardized protocols.

### TCRβ sequencing and analysis

RNA extracted from PBMCs, tumor biopsies, and TIL products using standard methods were used for the high-throughput sequencing of the TCRβ CDR3 region. Library preparation and sequencing was performed at CeGaT (Tübingen, Germany). Sequence reads were mapped, clonotypes were identified based on nucleotide and amino acid identity, and their frequencies calculated.

Clonotypes detected in the infused TIL product were defined as “TIL-derived.” Metrics including clonality (1–Pielou’s evenness), Shannon diversity index and D50 were calculated using established methods. TCR similarity was quantified using the Morisita–Horn index, which ranges from 0 (no overlap) to 1 (identical repertoires) (16). Longitudinal tracking of TIL-derived and endogenous clonotypes was performed across all timepoints. Expansion and contraction dynamics of TCR clonotypes between samples were analyzed using a two-sided binomial test with Bonferroni correction, with significance set at P < 0.01 (19).

Pharmacokinetic parameters—Cmax (peak frequency), Tmax (time to peak), and AUC (area under the curve)—were computed for each TIL infusion based on clonal frequency trajectories. Inter-compartmental overlap and clonotype sharing were assessed between tumor, blood, and TIL compartments.

## Case presentation

**Figure 1:**
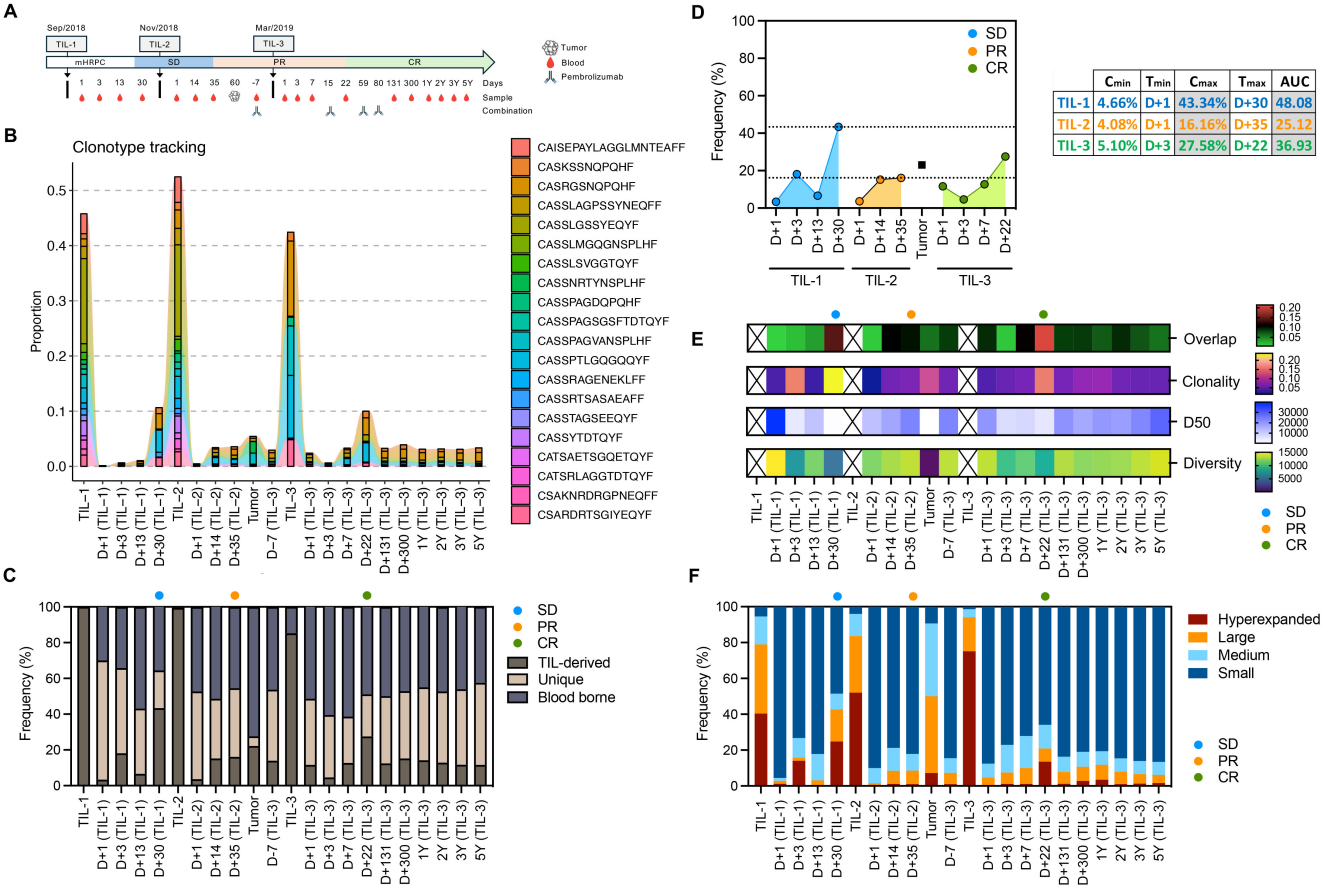
Longitudinal tracking of TIL-derived clonotypes, diversity, and clonal expansion following serial TIL infusions and anti-PD-1 therapy. **(A)** Timeline of treatments, clinical events (SD, stable disease; PR, partial response; CR, complete response), and serial blood/tumor sample collection. **(B)** Tracking of the top 20 most frequent clonotype from TIL-1 infusion across different samples. **(C)** Proportion of TIL-derived, unique, and blood-borne clonotypes over time, illustrating durable engraftment and tracking of infused TILs. **(D)** Pharmacokinetic parameters (Cmin, Tmax, Cmax, AUC) for TIL-derived clonotypes across TIL-1, TIL-2, and TIL-3 infusions, quantifying magnitude and kinetics of TIL engraftment and expansion. **(E)** Clonality, D50 (number of clones comprising 50% of the repertoire), diversity, and overlap between blood and tumor across multiple timepoints, from pre-treatment to 5 years post-infusion. Infused TIL products were omitted from this panel to improve visualization of repertoire dynamics across blood and tumor samples. **(F)** Distribution of TCR clonotypes classified as hyperexpanded (>1% of the repertoire), large (0.1–1%), medium (0.01–0.1%), or small (<0.01%) in blood and tumor samples, illustrating peaks of clonal expansion following TIL infusions and subsequent anti–PD-1 therapy.

The patient was a male in his 70s diagnosed with mCRPC characterized by rapid clinical progression and poor response to prior standard treatments, including androgen deprivation therapy, docetaxel chemotherapy, and second-line hormonal agents (18). A total of three autologous TIL infusions (TIL-1, TIL-2, TIL-3) were administered over a span of six months, with each infusion followed by systematic blood and tumor sampling (**Figure 1A**). TILs were expanded from a soft tissue metastasis biopsy, followed by ex vivo rapid expansion using anti-CD3/CD28 stimulation and irradiated feeder cells under GMP conditions. No enrichment for antigen specificity was performed. The infused products consisted of unselected CD3+ lymphocytes, with infusion doses ranging from 1.4 × 10^9^ to 8.0 × 10^9^ total T cells per treatment. Each infusion was preceded by a single-dose intravenous cyclophosphamide (60 mg/kg) and followed by low-dose IL-2 support (600,000 IU/kg, 1–5 doses depending on tolerance).

Following the first TIL infusion, the patient experienced disease stabilization (SD). A second infusion was given six weeks later, after which a post-treatment biopsy was performed on Day 60 to evaluate tumor infiltration. Imaging studies at this time demonstrated a partial response (PR). The third infusion, administered approximately six months after the first, was combined with pembrolizumab (200 mg IV every 3 weeks for four doses) starting on the day of infusion. This decision was based on the presence of residual disease and aimed to enhance T-cell function and prevent immune escape. Complete response (CR) was subsequently confirmed by imaging and maintained throughout a five-year follow-up period.

Peripheral blood samples were collected at baseline, following each TIL infusion, and periodically during long-term follow-up. TCRβ sequencing was performed on peripheral blood mononuclear cells (PBMCs), the infused TIL products, and tumor biopsies (pre-treatment and post-TIL-2) (16). TCR repertoire metrics—including clonality, diversity (Shannon index), D50, and inter-sample overlap (14, 16) —were used to track the fate and dynamics of TIL-derived, blood-borne, and novel clonotypes over time.

## Results

To assess the clonal dynamics and persistence of TIL-derived clonotypes, we performed high-throughput TCR sequencing on serial blood and tumor samples collected before and after three TIL infusions and anti-PD-1 therapy (15, 16, 18). Sample collection spanned a five-year period and aligned with major clinical interventions and milestones (**Figure 1A**) (18).

We tracked the frequency of the top 20 clonotypes from TIL-1 infusion across different samples and timepoints. Remarkably, no TIL-derived clonotypes were detected in the baseline blood sample, confirming that all tracked clonotypes originated from the infused product. Each TIL infusion was followed by clonal expansion events in the blood, reflected by increased clonality and decreased diversity, consistent with transient proliferation of dominant clonotypes (**Figure 1B**). These expansion patterns mirrored clinical improvement, suggesting that the magnitude of TIL-derived clonal expansion reflects therapeutic response (13). To confirm the clonotype dynamics and long-term persistence, we performed full TCR overlap analyses between samples and classified each clonotype as TIL-derived (present in the infused TIL product but absent at baseline blood sample), unique (detected only in a given sample), or blood-borne (shared across blood samples but not TIL-derived). This approach enabled mapping of the frequency of all TIL-derived clonotypes in each compartment and confirmed their expansion patterns as well as their durable persistence throughout follow-up. (**Figure 1C**). This stability reinforces the concept of durable engraftment of transferred TILs and ongoing immunologic surveillance (2, 15, 17). Notably, clonotypes from the first TIL product showed long-term persistence, supporting sustained immune engagement.

Additionally, dominant TIL-derived clonotypes present in the infused TIL product were subsequently identified the tumor biopsy collected two months post second infusion (**Figures 1B, C**), providing some evidence that successful TIL infiltration into the tumor microenvironment contributed to the observed clinical response. The temporal association between peaks in TIL-derived clonal expansion and clinical improvement (SD to PR to CR) further underscores the role of TIL tumor infiltration in achieving sustained therapeutic benefit (13).

The second infusion (TIL-2) induced a modest expansion, while the third infusion (TIL-3), co-administered with pembrolizumab, was associated with a renewed and delayed peak in TIL-derived clonotypes (**Figure 1C**).

Pharmacokinetic analysis of TIL-derived clonotypes—measured using peak frequency (Cmax), time to peak (Tmax), and area under the curve (AUC)—demonstrated that TIL-1 had the highest initial clonal expansion, while TIL-3 produced a delayed but substantial response, temporally aligned with PD-1 blockade (**Figure 1D**). TIL-2 showed a lower AUC overall, possibly due to insufficient activation or rapid contraction. These trends suggest that repeated TIL infusions can elicit variable expansion profiles and that immune checkpoint blockade may potentiate secondary clonal proliferation. The sustained detection of TIL-derived clonotypes at high frequency long after each infusion, particularly following repeated dosing, demonstrates that serial TIL therapy could promote prolonged engraftment and persistence, as demonstrated in CAR-T therapy (14, 23).

We further analyzed the structure of the TCR repertoire to identify shifts in clonal dominance. During periods of maximal expansion, the repertoire was characterized by an increase in overlap between blood/tumor sample and TIL product, higher clonality and frequency of hyperexpanded clones, with a corresponding reduction in smaller clonal populations resulting in reduced TCR diversity (**Figures 1E, F**). These periods coincided with transitions from SD to PR and later to CR, supporting the notion that therapeutic efficacy was associated with a narrowed but more tumor-specific TCR repertoire (15). Although this pattern aligned with radiographic response transitions, causality cannot be firmly established in a single-patient study.

**Figure 2:**
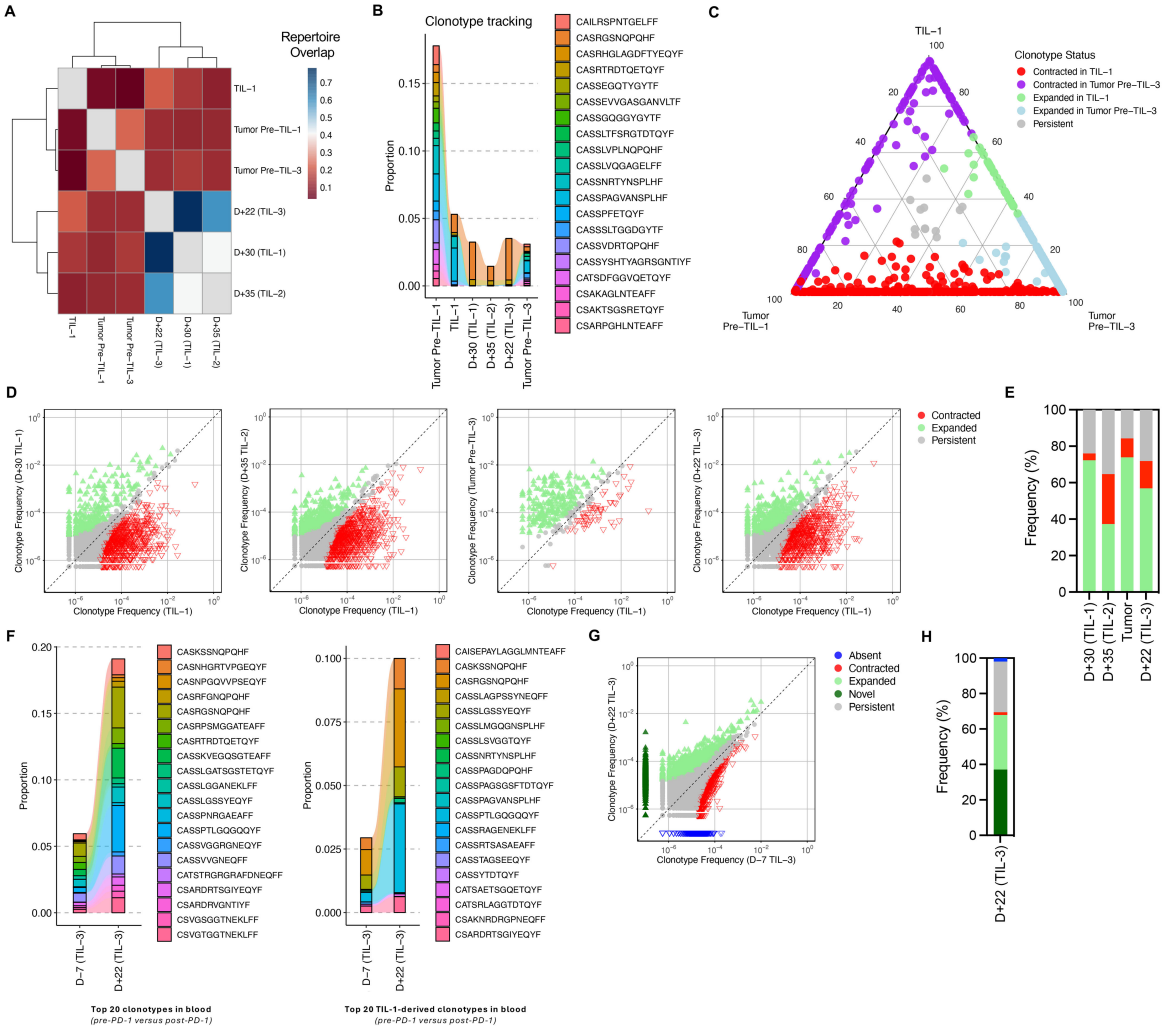
Clonotype sharing and anti-PD-1–mediated expansion of TIL-derived and novel clonotypes. **(A)** Clustering of samples based on TCR overlap, calculated using the Morisita–Horn index. This index ranges from 0 to 1, with 0 indicating private repertoires (no shared clonotypes) and 1 indicating public repertoires (identical clonotypes). **(B)** Tracking of the top 20 clonotypes present in the pre-TIL tumor sample across TIL-1 product, blood samples and tumor sample post-TIL infusion. “Tumor Pre–TIL-1” refers to the biopsy collected at the same time as the sample used for TIL manufacturing, while “Tumor Pre–TIL-3” refers to the biopsy collected after the second TIL infusion. **(C)** Overlap of dominant TCR clonotypes between pre-TIL tumor, infused TIL product, and post-TIL tumor, demonstrating infiltration and persistence of TIL-derived clonotypes within the tumor microenvironment. The numbers on the ternary plot axes represent the relative proportion of each clonotype within the three samples—Tumor Pre–TIL-1, TIL-1, and Tumor Pre–TIL-3. Each point reflects one clonotype, positioned according to its normalized abundance across the samples. **(D)** Scatterplots comparing TCR clone frequencies pre- and post-TIL treatment. Clones that were significantly expanded (green) or contracted (red) post-treatment based on a binomial test (two-sided, Bonferroni corrected P value < 0.01) are highlighted. **(E)** Bar plot of fraction of clones based on their status as quantified from panel **(D, F)** Frequency of top 20 TIL-1–derived clonotypes in blood pre- and post-anti-PD-1, and distribution of these clonotypes within tumor samples post-TIL therapy and anti-PD-1 treatment, illustrating the amplification and diversification of anti-tumor immune responses. **(G)** Scatterplot comparison TCR clonotypes in blood samples before and after anti-PD-1 therapy, showing expansion of both TIL-derived and novel clonotypes. **(H)** Bar plot of fraction of clones based on their status.

To evaluate tumor infiltration, we examined the overlap between dominant TCR clonotypes in the infused TIL product and post-infusion tumor biopsy. A subset of these clonotypes was detected in the tumor two months after the second infusion, suggesting trafficking and persistence in the tumor microenvironment (**Figures 2A–C**). Clonotypes originally identified in the pre-treatment tumor were found across compartments—infused TILs, blood, and post-treatment tumor—indicating broad systemic circulation and potential re-infiltration (**Figures 2B, C**). The clonotypes exhibited dynamic patterns of expansion and contraction between compartments, overall demonstrating *in vivo* expansion. These findings support the notion that tumor-derived T-cells not only expand systemically but also effectively home back to the tumor site and contribute to anti-tumor activity (15, 17). This is further supported by the observation that the most dominant expanding clonotypes coincided with clinical improvement (**Figures 2D, E**).

Following anti-PD-1 therapy, we observed expansion of both infused and previously undetected TCR clonotypes (**Figures 2F–H**). Among the top 20 clonotypes derived from TIL-1, several exhibited renewed expansion post-pembrolizumab and were also present in post-treatment tumor tissue. These findings support the hypothesis that PD-1 blockade may reinvigorate exhausted TIL-derived T cells and enhance both the proliferation and tumor-homing capacity of effective T-cell clones (20) as well promote the emergence of new anti-tumor clonotypes (19), as previously shown in other solid tumors.

## Discussion

Together, these findings provide strong evidence that multiple adoptive TIL dosing can induce durable immune reprogramming, while delayed PD-1 blockade can reawaken and amplify this response, promoting both persistence and diversification of tumor-reactive T-cells. This case provides longitudinal evidence for the durable engraftment, expansion, and reactivation of TIL-derived clonotypes following adoptive cell transfer and subsequent PD-1 blockade (18). Serial TCR sequencing revealed that peaks of TIL-derived clonal expansion were temporally associated with clinical response and ongoing disease control.

Importantly, anti-PD-1 therapy administered months after TIL infusion led to a robust secondary expansion of both original TIL-derived and novel clonotypes, resulting in durable complete response and suggesting that checkpoint blockade can reinvigorate transferred clones and promote new tumor-reactive responses (19, 20). These findings support the use of sequential or combination immunotherapies, as reinforced by recent translational studies (14, 15, 17, 21).

A recent phase 2 trial in gastrointestinal cancers showed that combining neoantigen-selected TILs with pembrolizumab increased the ORR from 7.7% to 23.5% (17),. These tumor responses are notable given the relatively low tumor mutational burden (TMB) in these cancers, as TMB has been associated with response to TIL therapy (26, 27). This demonstrates that TILs, particularly when combined with checkpoint blockade, can elicit activity even in low-mutation cancers traditionally considered less responsive to immunotherapy. Moreover, the combination of TIL with anti–PD-1 therapy achieved a 23% ORR in NSCLC patients who were previously resistant to PD-1 therapy (28), indicating that combinatory therapies can be effective across a broad range of malignancies. Here, through longitudinal TCR tracking, we demonstrate durable persistence and reactivation of TIL-derived tumor-reactive clonotypes after adoptive TIL transfers combined with anti–PD-1 therapy, supporting prolonged clinical remission.

Amaria et al. reported a lack of objective responses in recurrent or refractory ovarian cancer, colorectal cancer, and pancreatic ductal adenocarcinoma patients treated with TIL therapy (6). Although no responses were observed, 63% of patients achieved stable disease with evidence of antitumor activity. These findings suggest that multiple administrations or combination strategies may be required to enhance TIL efficacy and induce responses in immunologically “cold” tumors that are otherwise difficult to treat with a single infusion. Consistent with this, we have recently shown that serial TIL infusions promoted tumor infiltration of TIL-derived CD8^+^ clonotypes and led to complete disease remission in glioblastoma (22), indicating that repeated dosing may improve the pharmacokinetics and pharmacodynamics of TIL therapy and help drive clinical responses.

mCRPC patients are generally refractory to ICB (29, 30) due to the presence of exhausted and dysfunctional T-cells (31), a low TMB (32), and an immunosuppressive microenvironment enriched in myeloid cells that dampen T-cell responses (33). These immune evasion mechanisms may be overcome by modulating myeloid–T-cell interactions (31) or through combinatorial immunotherapies (34). Here we demonstrate that combining polyclonal TILs with ICB could reshape the tumor microenvironment, enable effective immune responses, and achieve complete disease remission.

While our observations suggest a temporal association between TIL-derived clonal expansion and clinical improvement, this relationship should be interpreted cautiously given the single-patient nature of the study. The patterns of clonality and diversity did not consistently align with each infusion, highlighting the complexity of immune dynamics in adoptive therapy. This may reflect saturation of dominant clones, exhaustion, or homeostatic mechanisms limiting further expansion. Additionally, while the blood and tumor repertoires shared several dominant clonotypes, they also showed notable divergence, particularly after TIL-3. This likely reflects distinct selective pressures in circulation versus the tumor microenvironment, or differences in sampling depth. The tumor biopsy obtained prior to TIL-3 was collected at a later timepoint than the blood samples shown in **Figure 2B**, and should be interpreted in that context.

Several clonotypes were shared between the baseline tumor (Tumor Pre–TIL-1) and the post-TIL tumor collected before the third infusion (Tumor Pre–TIL-3). However, the most frequent clonotypes in the initial tumor markedly decreased in the later sample, indicating clonal remodeling between lesions. This pattern suggests that, in addition to the persistence of infused TIL-derived clonotypes, new endogenous T-cell populations may have been recruited or expanded in response to evolving tumor antigens.

This longitudinal case study illustrates the complex immunologic dynamics associated with repeated TIL infusions and delayed PD-1 blockade in a patient with metastatic prostate cancer. We observed detectable persistence of TIL-derived clonotypes in peripheral blood for up to five years, with dynamic patterns of clonal expansion and contraction following each infusion. Notably, the combination of adoptive cell therapy with subsequent checkpoint inhibition was temporally associated with re-expansion of previously infused clonotypes and emergence of novel TCRs.

While these findings suggest that sequential TIL dosing and delayed PD-1 therapy may support durable antitumor immunity, they must be interpreted within the context of a single-patient study. Nevertheless, our data highlight the value of longitudinal TCR tracking to evaluate the pharmacokinetics, biodistribution, and activation of infused lymphocytes in adoptive immunotherapy. Future studies incorporating multi-patient cohorts and functional assays are warranted to further define the mechanisms underlying durable responses.

## Data Availability

The datasets presented in this study can be found in online repositories. The names of the repository/repositories and accession number(s) can be found below: https://www.ncbi.nlm.nih.gov/geo/, GSE295713.

## References

[1] Martín-Lluesma S Svane IM Dafni U Vervita K Karlis D Dimopoulou G. Efficacy of TIL therapy in advanced cutaneous melanoma in the current immuno-oncology era: updated systematic review and meta-analysis. Ann Oncol. (2024) 35:860–72. doi: 10.1016/j.annonc.2024.07.723, PMID: 39053767

[2] Schoenfeld AJ Lee SM Doger de Spéville B Gettinger SN Häfliger S Sukari A. Lifileucel, an autologous tumor-infiltrating lymphocyte monotherapy, in patients with advanced non–small cell lung cancer resistant to immune checkpoint inhibitors. Cancer Discov. (2024) 14:1389–402. doi: 10.1158/2159-8290.CD-23-1334, PMID: 38563600 PMC11294887

[3] Jazaeri AA Zsiros E Amaria RN Artz AS Edwards RP Wenham RM. Safety and efficacy of adoptive cell transfer using autologous tumor infiltrating lymphocytes (LN-145) for treatment of recurrent, metastatic, or persistent cervical carcinoma. J Clin Oncol. (2019) 37:2538–8. doi: 10.1200/JCO.2019.37.15_suppl.2538

[4] O’Malley D Lee S Psyrri A Sukari A Thomas S Wenham R. 492 Phase 2 efficacy and safety of autologous tumor-infiltrating lymphocyte (TIL) cell therapy in combination with pembrolizumab in immune checkpoint inhibitor-naïve patients with advanced cancers. In: Regular and Young Investigator Award Abstracts. BMJ Publishing Group Ltd. J Clin Oncol. (2021). p. A523–4.

[5] Zacharakis N Huq LM Seitter SJ Kim SP Gartner JJ Sindiri S. Breast cancers are immunogenic: immunologic analyses and a phase II pilot clinical trial using mutation-reactive autologous lymphocytes. J Clin Oncol. (2022) 40:1741–54. doi: 10.1200/JCO.21.02170, PMID: 35104158 PMC9148699

[6] Amaria R Knisely A Vining D Kopetz S Overman MJ Javle M. Efficacy and safety of autologous tumor-infiltrating lymphocytes in recurrent or refractory ovarian cancer, colorectal cancer, and pancreatic ductal adenocarcinoma. J Immunother Cancer. (2024) 12:1–11. doi: 10.1136/jitc-2023-006822, PMID: 38309721 PMC10840042

[7] Pedersen M Westergaard MCW Milne K Nielsen M Borch TH Poulsen LG. Adoptive cell therapy with tumor-infiltrating lymphocytes in patients with metastatic ovarian cancer: a pilot study. Oncoimmunology. (2018) 7:e1502905. doi: 10.1080/2162402X.2018.1502905, PMID: 30524900 PMC6279323

[8] Kverneland AH Pedersen M Westergaard MCW Nielsen M Borch TH Olsen LR. Adoptive cell therapy in combination with checkpoint inhibitors in ovarian cancer. Oncotarget. (2020) 11:2092–105. doi: 10.18632/oncotarget.27604, PMID: 32547707 PMC7275789

[9] Amaria RN Komanduri KV Schoenfeld AJ Ramsingh G Burga RA Jagasia MH. Entering a new era of tumor-infiltrating lymphocyte cell therapy innovation. Cytotherapy. (2025) 27:864–73. doi: 10.1016/j.jcyt.2024.12.010, PMID: 40131263

[10] Navarro Rodrigo B Ortiz Miranda Y Corria-Osorio J Coukos G Harari A. Immune correlates and mechanisms of TIL therapy efficacy: current insights and knowledge gaps. Trends Cancer. (2025) 11. doi: 10.1016/j.trecan.2025.08.002, PMID: 40885613

[11] Creelan BC He K Garon E Chesney J Lee S Nieva J. 1488 A multicenter phase 2 trial of lifileucel plus pembrolizumab in patients with checkpoint inhibitor-naive metastatic NSCLC: updated results. In: Late-Breaking Abstracts. BMJ Publishing Group Ltd. J Immunother Cancer. (2024). p. A1718–8.

[12] Zhou X Wu J Duan C Liu Y. Retrospective analysis of adoptive TIL therapy plus anti-PD1 therapy in patients with chemotherapy-resistant metastatic osteosarcoma. J Immunol Res. (2020) :2020:1–12. doi: 10.1155/2020/7890985, PMID: 33062726 PMC7547340

[13] Albarrán Fernández V Ballestín Martínez P Stoltenborg Granhøj J Borch TH Donia M Marie Svane I. Biomarkers for response to TIL therapy: a comprehensive review. J Immunother Cancer. (2024) 12:e008640. doi: 10.1136/jitc-2023-008640, PMID: 38485186 PMC10941183

[14] Kirouac DC Zmurchok C Morris D. Making drugs from T cells: The quantitative pharmacology of engineered T cell therapeutics. NPJ Syst Biol Appl. (2024) 10:31. doi: 10.1038/s41540-024-00355-3, PMID: 38499572 PMC10948391

[15] Chiffelle J Barras D Pétremand R Orcurto A Bobisse S Arnaud M. Tumor-reactive T cell clonotype dynamics underlying clinical response to TIL therapy in melanoma. Immunity. (2024) 57:2466–82. doi: 10.1016/j.immuni.2024.08.014, PMID: 39276771

[16] Chiffelle J Genolet R Perez MA Coukos G Zoete V Harari A. T-cell repertoire analysis and metrics of diversity and clonality. Curr Opin Biotechnol. (2020) 65:284–95. doi: 10.1016/j.copbio.2020.07.010, PMID: 32889231

[17] Lowery FJ Goff SL Gasmi B Parkhurst MR Ratnam NM Halas HK. Neoantigen-specific tumor-infiltrating lymphocytes in gastrointestinal cancers: a phase 2 trial. Nat Med. (2025) 31. doi: 10.1038/s41591-025-03627-5, PMID: 40169866 PMC12315913

[18] Karbach J Kiselicki D Brand K Wahle C Sinelnikov E Gustavus D. Tumor-infiltrating lymphocytes mediate complete and durable remission in a patient with NY-ESO-1 expressing prostate cancer. J Immunother Cancer. (2023) 11:e005847. doi: 10.1136/jitc-2022-005847, PMID: 36627144 PMC9835940

[19] Yost KE Satpathy AT Wells DK Qi Y Wang C Kageyama R. Clonal replacement of tumor-specific T cells following PD-1 blockade. Nat Med. (2019) 25:1251–9. doi: 10.1038/s41591-019-0522-3, PMID: 31359002 PMC6689255

[20] Wei SC Levine JH Cogdill AP Zhao Y Anang NAAS Andrews MC. Distinct cellular mechanisms underlie anti-CTLA-4 and anti-PD-1 checkpoint blockade. Cell. (2017) 170:1120–33. doi: 10.1016/j.cell.2017.07.024, PMID: 28803728 PMC5591072

[21] Arruda LC Karbach J Kiselicki D Altmannsberger H Sinelnikov E Gustavus D. Multiple infusions of TILs expanded with IL-2/IL-15/IL-21 lead to an improved PK/PD profile and long-term clinical responses in patients with cold tumors. Cytotherapy. (2025) 27:S165. doi: 10.1016/j.jcyt.2025.03.331

[22] Arruda LCM Karbach J Kiselicki D Altmannsberger HM Sinelnikov E Gustavus D. Tumor-infiltrating lymphocytes-derived CD8 ^+^ clonotypes infiltrate the tumor tissue and mediate tumor regression in glioblastoma. Oncoimmunology. (2025) 14:1–16. doi: 10.1080/2162402X.2025.2559784, PMID: 41054926 PMC12505509

[23] Liu Y Deng B Hu B Zhang W Zhu Q Liu Y. Sequential different B-cell antigen–targeted CAR T-cell therapy for pediatric refractory/relapsed Burkitt lymphoma. Blood Adv. (2022) 6:717–30. doi: 10.1182/bloodadvances.2021004557, PMID: 34521107 PMC8945318

[24] Richardson T Holtick U Frenking JH Tharmaseelan H Balke-Want H Flümann R. Sequential BCMA CAR T-cell therapy in refractory multiple myeloma. Blood Adv. (2025) 9:4624–30. doi: 10.1182/bloodadvances.2025016712, PMID: 40644619 PMC12455134

[25] Meng Y Deng B Rong L Li C Song W Ling Z. Short-interval sequential CAR-T cell infusion may enhance prior CAR-T cell expansion to augment anti-lymphoma response in B-NHL. Front Oncol. (2021) 11. doi: 10.3389/fonc.2021.640166, PMID: 34277400 PMC8279746

[26] Lauss M Donia M Harbst K Andersen R Mitra S Rosengren F. Mutational and putative neoantigen load predict clinical benefit of adoptive T cell therapy in melanoma. Nat Commun. (2017) 8:1738. doi: 10.1038/s41467-017-01460-0, PMID: 29170503 PMC5701046

[27] Levi ST Copeland AR Nah S Crystal JS Ivey GD Lalani A. Neoantigen identification and response to adoptive cell transfer in anti–PD-1 naïve and experienced patients with metastatic melanoma. Clin Cancer Res. (2022) 28:3042–52. doi: 10.1158/1078-0432.CCR-21-4499, PMID: 35247926 PMC9288495

[28] Creelan BC Wang C Teer JK Toloza EM Yao J Kim S. Tumor-infiltrating lymphocyte treatment for anti-PD-1-resistant metastatic lung cancer: a phase 1 trial. Nat Med. (2021) 27:1410–8. doi: 10.1038/s41591-021-01462-y, PMID: 34385708 PMC8509078

[29] Powles T Yuen KC Gillessen S Kadel EE Rathkopf D Matsubara N. Atezolizumab with enzalutamide versus enzalutamide alone in metastatic castration-resistant prostate cancer: a randomized phase 3 trial. Nat Med. (2022) 28:144–53. doi: 10.1038/s41591-021-01600-6, PMID: 35013615 PMC9406237

[30] Beer TM Kwon ED Drake CG Fizazi K Logothetis C Gravis G. Randomized, double-blind, phase III trial of ipilimumab versus placebo in asymptomatic or minimally symptomatic patients with metastatic chemotherapy-naive castration-resistant prostate cancer. J Clin Oncol. (2017) 35:40–7. doi: 10.1200/JCO.2016.69.1584, PMID: 28034081

[31] Kfoury Y Baryawno N Severe N Mei S Gustafsson K Hirz T. Human prostate cancer bone metastases have an actionable immunosuppressive microenvironment. Cancer Cell. (2021) 39:1464–1478.e8. doi: 10.1016/j.ccell.2021.09.005, PMID: 34719426 PMC8578470

[32] Yarchoan M Hopkins A Jaffee EM. Tumor mutational burden and response rate to PD-1 inhibition. New Engl J Med. (2017) 377:2500–1. doi: 10.1056/NEJMc1713444, PMID: 29262275 PMC6549688

[33] Lyu A Fan Z Clark M Lea A Luong D Setayesh A. Evolution of myeloid-mediated immunotherapy resistance in prostate cancer. Nature. (2025) 637:1207–17. doi: 10.1038/s41586-024-08290-3, PMID: 39633050 PMC11779626

[34] Lu X Horner JW Paul E Shang X Troncoso P Deng P. Effective combinatorial immunotherapy for castration-resistant prostate cancer. Nature. (2017) 543:728–32. doi: 10.1038/nature21676, PMID: 28321130 PMC5374023

